# Electrocution as an alternative euthanasia method to blunt force trauma to the head followed by exsanguination for non-viable piglets

**DOI:** 10.1186/s13028-020-00565-9

**Published:** 2020-12-07

**Authors:** Johannes Husheer, Matthias Luepke, Peter Dziallas, Karl-Heinz Waldmann, Alexandra von Altrock

**Affiliations:** 1 Clinic for Swine, Small Ruminants, Forensic Medicine and Ambulatory Service, University of Veterinary Medicine Hannover, Foundation, 30173 Hannover, Germany; 2 Department of General Radiology and Medical Physics, University of Veterinary Medicine Hannover, Foundation, 30173 Hannover, Germany; 3 Clinic for Small Animals, University of Veterinary Medicine Hannover, Foundation, 30559 Hannover, Germany

**Keywords:** Cardiac arrest, Electrical stunning, Finite element analysis, Isoelectric EEG, Ventricular fibrillation

## Abstract

**Background:**

On farms, the currently approved and most widely practised method of euthanising non-viable piglets is blunt force trauma to the head followed by exsanguination. However, the use of this method is criticised due to public perceptions and aversion to the methodology by caretakers. Therefore, electrocution after electrical stunning was examined as an alternative approach in 80 hybrid piglets. Initially, electrocution was simulated with finite element analysis using a computer piglet-model, where current density in the heart was visualised and size and position of the electrodes were defined. The following step investigated electrical parameters for electrocution in anaesthetised piglets; first, with a constant voltage power source and then with a constant current power source. The electrical stunning was examined using the constant current supply. Finally, the results of electrical stunning and electrocution were verified in 25 healthy piglets with a body weight between 1 and 2 kg. Unconsciousness was proven by testing palpebral, corneal and nociceptive reflexes. Time of death was confirmed by electroencephalography (EEG) and electrocardiography (ECG) records.

**Results:**

Stunning succeeded with the preset of 1.3 A and 50 Hz, placing the electrodes on both sides of the head between the eyes and ears using different timespans between 8 and 20 s. Prolonged electrical flow resulted in reduced paddling movements after the epileptic seizure, and allowed undisturbed reflex tests and installation of electrodes for EEG and ECG recording during electrocution. Using 0.75 A and 400 Hz, pin-shaped electrodes were first positioned on both sides of the chest for 5 s, followed by a break of 20–30 s and a second current flow, whereby the electrodes were placed above the withers and the sternum for 5 s. Cardiac arrest and an isoelectric EEG were induced within 3 min after the onset of the electrical flow through the chest. The most obvious indicator of effective stunning and electrocution was termination of rhythmic breathing. Piglets with cardiac arrest showed only single gasps lasting up to 3 min after electrocution.

**Conclusions:**

The evaluated stunning and electrocution protocol might ease concerns about timely piglet euthanasia. However, this should be verified in non-viable piglets to exclude influencing factors like dehydration and diseases.

## Background

Euthanasia is a necessary management tool for agricultural production of swine that provides a means to prevent individual animals from suffering. The search for humane methods to euthanise piglets is critical to address public concern [1]. A humane method induces minimal pain and distress to the pig during administration and a rapid loss of consciousness. Death must be achieved quickly and consistently. Additionally, it has to be economically and practically feasible in the context of the type of production; otherwise, it will not be used by the farmer [2]. The blunt force trauma to the head followed by exsanguination is a humane method for euthanasia especially for neonatal animals. This method is recommended in different federal states of Germany. Nonetheless, it is often aesthetically displeasing to some farm workers and to the public in general [3, 4]. For this reason, the search for alternatives is required [3].

Euthanasia by electrocution after electrical stunning is a very efficient method that is painless for the animal [4]. A current must pass through the pig’s brain to induce a grand mal epileptic seizure with insensibility, and then must cross the heart to induce cardiac fibrillation [3], this leading to circulatory collapse and instantaneous death. However, there are objections regarding the use of electrocution in small piglets (body weight < 5 kg) [5, 6] due to the assumption that ventricular fibrillation may not persist after cessation of current flow [3]. Nevertheless, literature validating this assumption is limited.

The objective of the present study was to determine whether electrocution of piglets with a body weight less than 2 kg is a humane and safe method of euthanasia which is in accordance with animal welfare legislation. For this purpose, the best contact points and sizes of electrodes, amperage, voltage, current frequency, duration of the current flow and shape of the electrodes were identified.

## Methods

### Part I: development of a computer piglet model

One clinically healthy male hybrid piglet (Landrace × Large White × Pietrain, body weight (BW): 2.01 kg, age: 5 days) was used to acquire computed tomography (CT)- and magnetic resonance imaging (MRI)-datasets in order to create a computer piglet model. The piglet was anaesthetised with azaperone (Stresnil® 2 mg/kg BW; Elanco, Bad Homburg, Germany) and ketamine (Ketamidor® 20 mg/kg BW, Wirtschaftsgenossenschaft deutscher Tierärzte e.G. (WDT), Garbsen, Germany) and maintained with ketamine. A saphenous vein catheter was placed and the piglet was positioned and fixed in ventral recumbency on a foam tube to obtain congruent images using CT and MRI.

Image acquisition was performed using a 64 multi-detector-row CT scanner (Brilliance CT 64—Philips GmbH, Hamburg, Germany). Transverse images of the whole thorax were acquired in an axial scan-mode using the following parameters: 120 kV, 500 mAs, 0.67 mm slice thickness and a square image matrix with size 512 × 512 pixels (pixel size 0.26 × 0.26 mm). Immediately thereafter, contrast media (14 mL of Xenetix® Guerbet, Gornchem, the Netherlands) was injected through the vein catheter and CT scans were repeated. Afterwards, the pig was euthanised using 40 mg/kg BW pentobarbital (Euthadorm®, CP-Pharma Handelsgesellschaft mbH, Burgdorf, Germany).

Still lying on the foam, images of the dead piglets’ organs were generated using magnet resonance imaging (MRI, Philips Archieva 3.0 T TX). The images were obtained using a T1 weighted sequence with 3D-TFE (voxel size 0.6 × 0.49 × 0.49 mm) and T2 weighted sequence with VISTA (voxel size 0.7 × 0.45 × 0.45 mm).

The program Amira® (FEI Technologies Inc., Hillsboro, OR, USA) was used for segmentation of the combined CT-MRI-datasets of the upper part of the body. The reconstructed transversal dataset was uploaded, and skin, lung, trachea, heart, vessels and bones were segmented by using manual slice-by-slice segmentation.

Triangular surface meshes of the segmented organs were created, and a volumetric tetrahedral grid was generated from the triangular surface using the software tool “Tetra Grid” (Amira®). The grid was minimised (DYNAmore FEM Ingenieurdienstleistungen GmbH, Stuttgart, Germany) and converted into NASTRAN format to import the data into the COMSOL Multiphysics® program (COMSOL AB, Stockholm, Sweden) for finite element (FE) analysis.

Based on the Database of Tissue Properties [7], which refers to human tissue, electrical conductivity was assigned to each organ. The simulation of the electric current flow through the heart was performed with different electrode sizes and positions while choosing corresponding numbers of triangles in the mesh. FE calculations were carried out with an alternating current (AC) voltage of 11.4 V. The course of the electrical current was visually displayed, and the current densities of the individual tissues of the model were calculated (current per area, [A/m^2^]).

### Validation of the FE analysis

To validate the FE analysis, the electrical currents flowing through the body were measured after an external voltage had been applied to the dead piglet. For this purpose, a cannula (18 G × 1 3/4, 1.3 × 45 mm, Vasofix Braunüle B. Braun Melsungen AG, Melsungen, Germany), which served as a recording electrode, was inserted dorsally through the skull into the brain. After confirming the position of the cannula in the brain by means of X-rays, flat electrodes were placed at the chosen positions, an AC voltage of 11.4 V was applied and the currents through the body were measured. Thereafter, the cannula was removed and inserted into the heart, penetrating the thorax on the left side close to the sternum behind the elbow between the rips. The flat electrodes were then placed at different positions on the piglet and the currents through the body were measured again. In addition, measurements were performed in which only two flat electrodes were placed on the skin. The voltage was checked with a multimeter (Bening MM1-3). The electrical currents through the body were read by another multimeter (Voltcraft VC 270) connected to the recording electrode. The same electrode positions and sizes were selected in the mesh of the 3D model for the FE analysis. The electrical currents were simulated at different electrode positions and compared with the measured values.

### Part II: examination of current parameters for electrocution and stunning of piglets

To define current parameters which induce cardiac fibrillation securely followed by cardiac arrest, 52 piglets (25 males, 27 females, mean age 2.7 ± 0.9 days, average BW 1.44 ± 0.44 kg) were included in this part of the study. Additionally, six piglets (three males, three females, including four piglets from the electrocution investigation) were used to determine a stunning protocol (mean age: 3.0 ± 1.41 days, average body weight: 1.63 ± 0.21 kg). All piglets came from a multiplier herd, producing hybrids based on crosses of Landrace × Large White × Pietrain. None of the piglets showed disorders of the general condition. Altogether, 17 piglets survived the trial.

### Examination of electrocution in anaesthetised piglet

Variations in current strength, frequency, duration of the current flow and shape of the electrodes were investigated to find a setting, which led to safe and immediate cardiac arrest. Piglets were anaesthetised with azaperone (Stresnil® 2 mg/kg; ELANCO, Bad Homburg, Germany) and ketamine (Ketamidor® 20 mg/kg, WDT, Garbsen, Germany) administered intramuscularly before the current flow was switched on. Anaesthesia was verified by the absence of palpebral and interdigital reflexes while the corneal reflex was still present.

Initially, a transformer was used to provide constant voltage using alternating current (AC), while the current strengths were read with a multimeter (Voltcraft VC 270) during the current flow. The anaesthetised piglets were laid in ventral recumbency. Based on the findings from the computer model, the electrodes were placed on both sides of the chest behind the elbows. Using electrode contact gel and a current frequency of 50 Hz, the duration of the current flow, the size of flat electrodes (2 × 2 cm, 3 × 3 cm) and the voltage were varied.

**Fig. 1:**
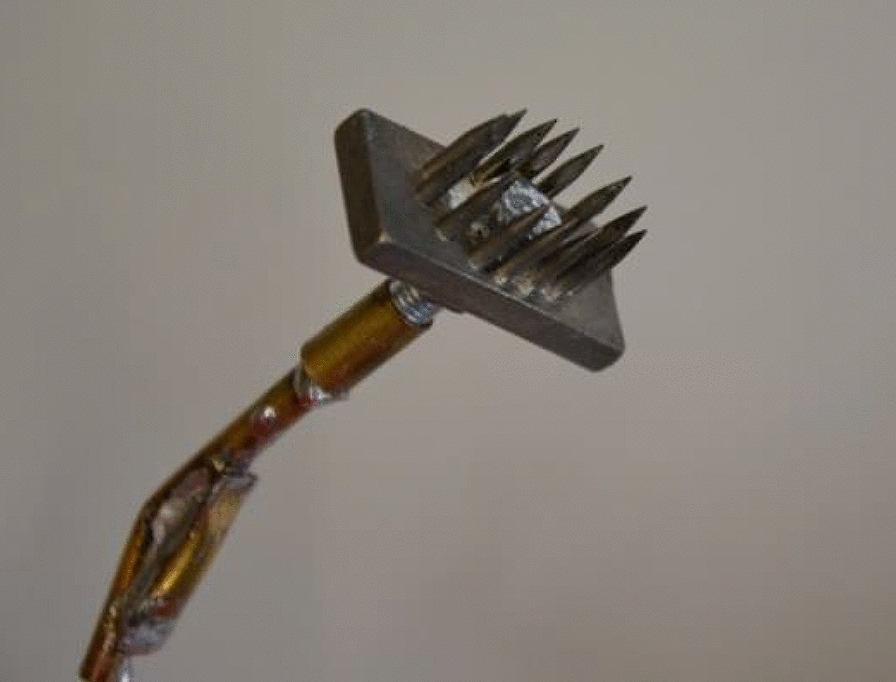
Pin-shaped electrodes (size: 1 × 2 cm, pin length: 1 cm)

Due to the lack of consistent successful electrocution using constant voltage, the current source was replaced by a high-frequency constant current electrical stunning transformer (STUN-E 514®, SCHROPP GmbH, Durach, Germany) in order to keep the current constant at 750 mA. Using AC and electrode contact gel, the frequency, duration of the current flow, shape of the electrodes [flat electrodes versus pin-shaped electrodes (size: 1 × 2 cm, pin length: 1 cm, Fig. 1)], and the position of the electrodes were changed. Additionally, a second electrical flow through the heart area was tested.

Principally, only one electrical parameter was changed after an unsuccessful attempt of electrical killing, and the setting was tested using the next piglet. The aim was to successfully euthanise five piglets one after the other with the same setting. Altogether, the authorities approved the use of 60 piglets at maximum for this part of the investigation.

### Evaluating the death of the piglets

The time of death was investigated 1, 3, 5, 7 and 10 min after the electrical flow. It was confirmed by using electrocardiography (ECG, EDAN® Veterinary PC EEG, VE-1010, EDAN Instruments Inc., Shenzhen, China), electroencephalography (EEG, Narcotrend®-Compact M, Narcotrend-Gruppe, Narcoscience GmbH & Co. KG, Hannover, Germany), and reflex tests.

For ECG recording, electrodes were fixed with clamps on both forelimbs and left rear limb and the neutral electrode on the right rear limb of the anaesthetised piglets. A conductivity gel was applied between the clamps and the skin to improve contact.

For EEG recording, disposable subdermal needle electrodes (steel, 0.4 × 13 mm, Klaus Schuler GmbH Medizintechnik, Freiburg, Germany) were placed under the skin on the skull, one in front of each ear, and the reference electrode in the midline just behind the eyes [8].

Records were read and interpreted according to the time schedule using a scoring system. The ECG was evaluated using three scores: score 0: sinus rhythm, score 1: cardiac fibrillation and score 2: cardiac arrest. The EEG pattern was examined for presence of an isoelectric EEG (yes/no), characterised as a flat line with very low-amplitude activities of the raw EEG and EEG stage (F) displayed on the Narcotrend® monitor.

Unconsciousness after electrocution was evaluated by the absence of palpebral and corneal reflexes as well as the absence of nociceptive reflexes by pinching the nasal septum and the interdigital web with surgical tweezers. Additionally, rhythmic breathing, gasping, vocalisation and movements without stimulation were recorded. The intensity of the movement was rated by using a score system: score 1: no movement, score 2: delayed, uncoordinated movement, and score 3: directed, pronounced movement.

Skin-irritations beneath the electrode contact surfaces were recorded after the last scoring and differentiated into mild (superficial, irregular skin burns smaller than the contact area of the electrodes), moderate (superficial skin burns covering the contact area of the electrodes) and severe (deep burns of the skin layer and deeper tissue).

### Examination of electrical stunning

In accordance with the Council Regulation (EC) No 1099/2009 of 24 September 2009 on the protection of animals at the time of killing, a recommended minimum current of 1.3 A was predefined for stunning using the high-frequency constant current electrical stunning transformer (STUN-E 514®, SCHROPP GmbH, Durach, Germany). The electrodes were placed between the eye and ear on each side of the head at the temples. Applying electrode contact gel, at first, flat electrodes (size: 2 × 2 cm) were used, but later replaced by pin-shaped electrodes (Fig. 1), based on the experiences acquired during the examination of the electrocution. Variations in current frequency and duration of the current flow were investigated to find a setting, which caused instantaneous loss of consciousness and a time interval of unconsciousness sufficient to enable reflex testing and placement of EEG and ECG electrodes before starting electrocution.

Immediately after electrical stunning, clinical signs of an epileptic seizure, i.e. immediate collapse, tonic and clonic phases, were recorded, and unconsciousness was verified by testing palpebral and corneal reflexes as well as the nociceptive reflexes at the nasal septum and the interdigital web as described for death evaluation. Additionally, vocalisation, rhythmic breathing, gasping and skin-irritations caused by the electrodes were recorded.

Following electrical stunning, electrodes of the EEG and ECG were fixed, as described above, and electrocution was performed using the successful setting gained in the first part of the investigation. The evaluation of the death followed the protocol as previously mentioned.

The aim was to stun five piglets successfully one after the other with the same setting.

### Part III: examination of the reproducibility of stunning and electrocuting in 25 piglets

For this part of the examination, 25 clinically healthy hybrid piglets (12 males, 13 females; breed: Landrace × Large White × Pietrain) were selected. Their body weight was between 1 and 2 kg (on average 1.46 ± 0.19 kg), the mean age was 2.32 ± 0.47 days. Based on the gained knowledge, the electrical parameters for stunning and electrocuting were applied to euthanise 25 piglets one after the other to verify the method as being effective for euthanasia. Unconsciousness and death were confirmed using the above mentioned protocol.

## Results

### Part I: simulation of electrocution using the 3D model of a piglet and validation of the FE analysis

**Fig. 2:**
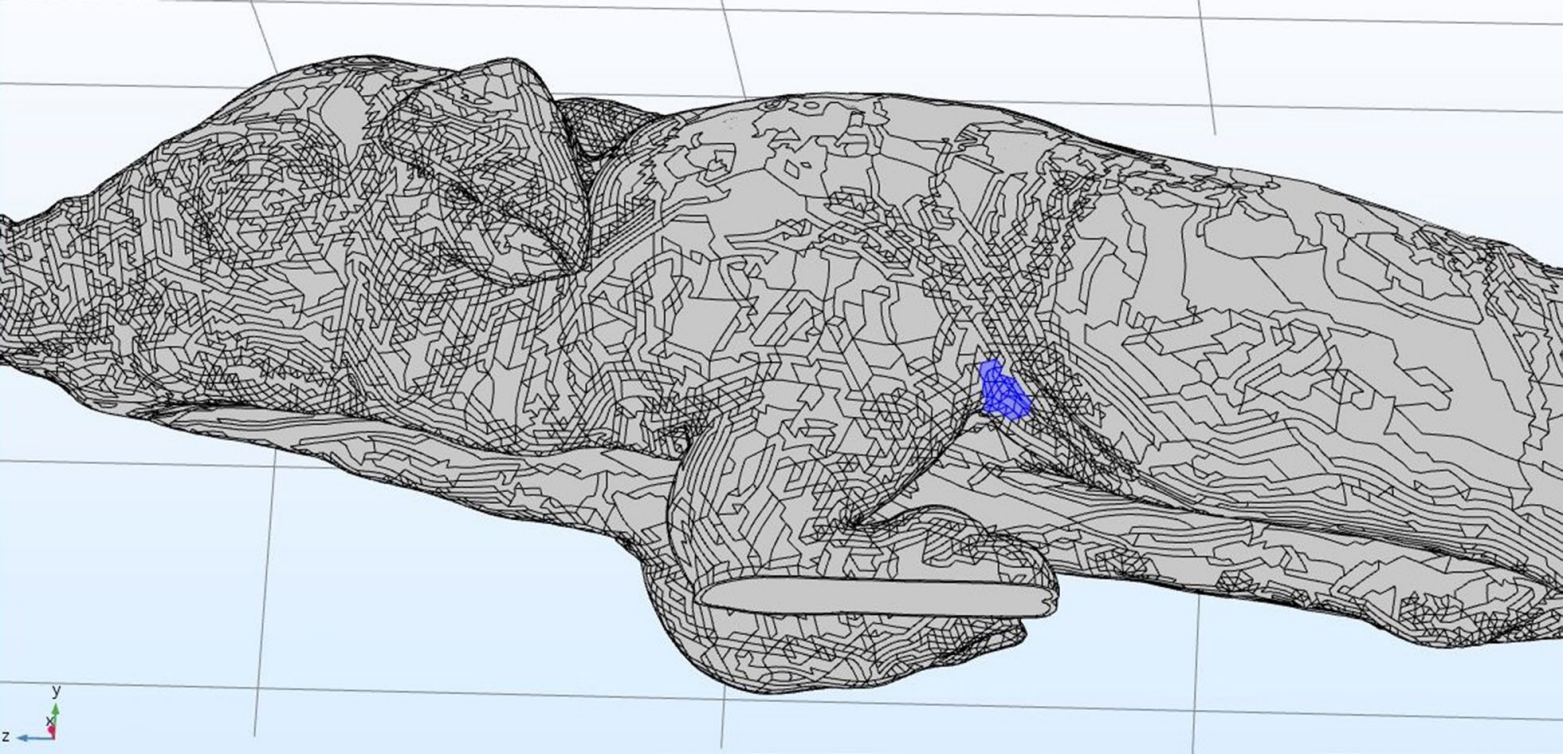
Volume meshes of the 3D-piglet model: Marked area of the electrodes position behind the elbow (blue) to simulate the path of electrical current through the heart

**Fig. 3:**
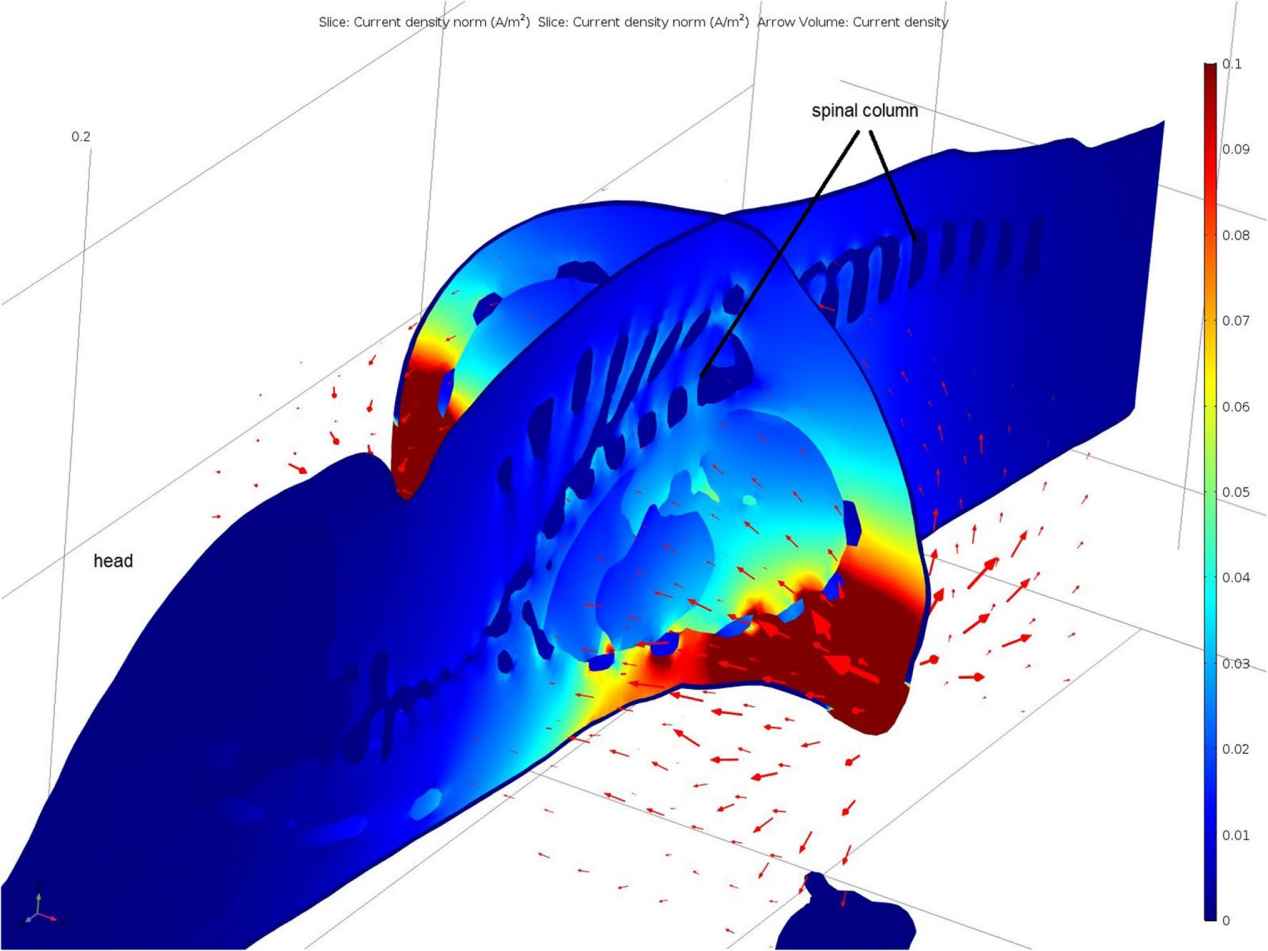
Simulation of the electrical flow through the chest of a piglet: electrodes placed on both sides behind the elbows; colour scale and arrow volume depict current density (A/m^2^)

**Fig. 4:**
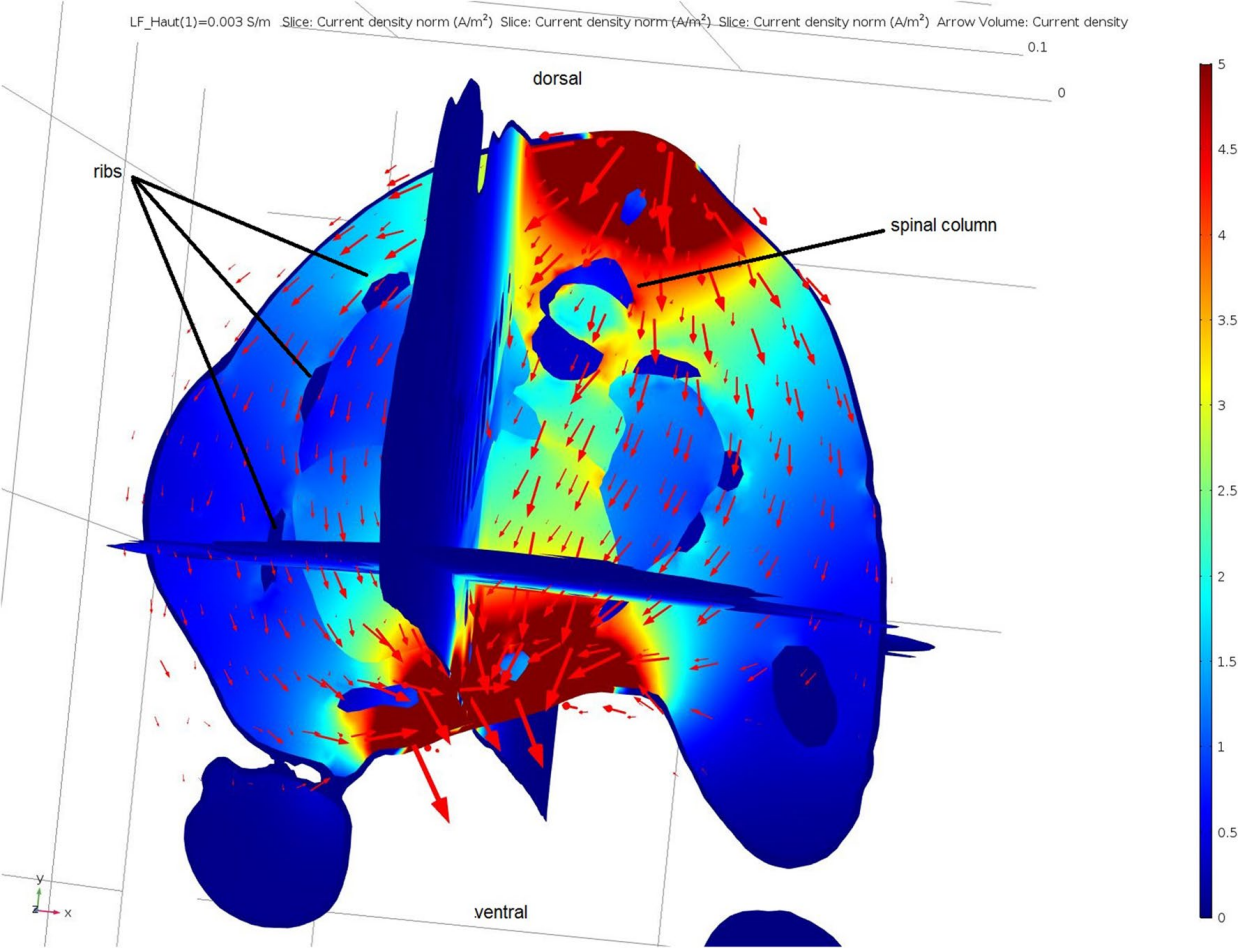
Simulation of the electrical flow through the chest of a piglet: electrodes placed above the sternum and the withers; colour scale and arrow volume depict current density (A/m^2^)

The electrical current flow through the heart was simulated using the constructed three-dimensional model of a piglet. The placement of electrodes could be simulated at any area of the surface mesh (Fig. 2). Two localisations were tested, placing the electrodes behind the elbows (Fig. 3) and above the sternum and the withers (Fig. 4). The course of the electrical current was visually displayed with arrows and the current densities of the individual tissues of the model were calculated, these being depicted in different colours.

The simulation showed a high current flow on the skin but also demonstrated the passing through the heart at a lower density (Figs. 3, 4). At the contact points of the electrodes, the highest current density occurred, this decreasing with increasing distance between the electrodes. The highest current density in the heart was obtained when the electrodes were placed behind the elbows with a current flow from side to side through the chest (Fig. 3).

For validation of the FE analysis, calculated currents were compared with measurements of electric current crossing the body and reaching a dead piglet´s heart and brain after applying an external voltage to two electrodes (Table 1). This indicates that the calculated current of the simulation was approximately two to ten times smaller than the measured current. The greatest deviations between simulation and measurement occurred when two flat electrodes were placed on the skin and the currents through the thorax were determined.

**Table 1:** Current strength between two electrodes at an applied voltage of 11.4 V (f = 50 Hz) testing different positions of electrodes

Placement of electrodes	Current strength (mA) through the body	
	Computer model	Dead piglet
On the left eye and in the brain	1.14	8.23
Between the left ear and eye and in the brain	1.06	5.27
On the left ear and in the brain	1.45	7.55
On both sides of the chest behind the elbows	0.71	11.33
Above the sternum and the withers	0.61	7.43
Above the withers and in the heart	1.07	2.73
Above the sternum and in the heart	1.15	4.67
On the left side of the chest behind the elbow and in the heart	2.27	3.50

### Part II: definition of electrical parameters for electrocution and electrical stunning

#### Electrocution of anaesthetised piglets with constant voltage

**Fig. 5:**
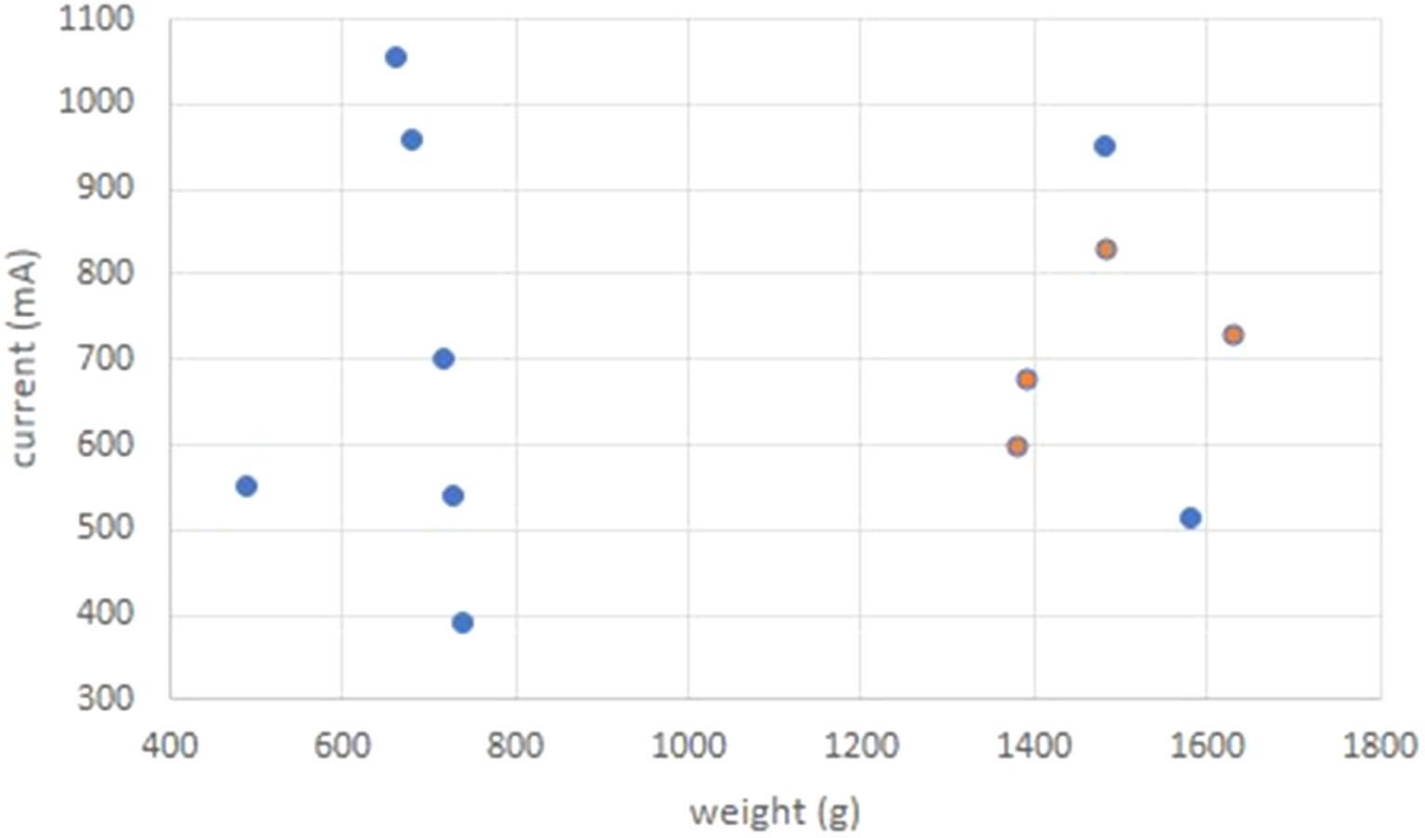
Weight and current strength 15 s after the onset of the electrical flow (red spots: piglets with cardiac arrest, blue spots: survivors)

Anaesthetised piglets (n = 13) were placed in sternal recumbency and electrodes for EEG and ECG recording were fixed. For electrocution, flat electrodes were placed on both sides of the chest behind the elbow. Starting with a voltage of 75 V and a duration of the electrical flow of 15 s, two piglets were successfully euthanised, while the third survived (Table 2). Afterwards, five different settings were tested, each on one piglet, changing only one electrical parameter from one setting to the next. Despite this, the piglets survived (Table 2). Based on the weight of the piglets and the success of the first setting, the approach was repeated with piglets heavier than 1.0 kg BW. Three piglets were successfully euthanised but the fourth survived. After the eighth setting, which also failed, the investigation was terminated. The analysis of the current strength during the electrical flow showed that the electrocution succeeded when the current strength reached 600–830 mA 15 s after the onset in piglets with a body weight between 1.38 and 1.63 kg (Fig. 5). Therefore, it was assumed that using an amperage-regulated circuit preselecting 750 mA could be successful in piglets with a body weight of approximately 1.50 kg.

**Table 2:** Experimental settings for electrocution of anaesthetised piglets (n = 13) using constant voltage of 50 Hz and flat electrodes placed on both sides of the chest behind the elbow

Setting	Number of successfully electrocuted piglets	Weight of the electrocuted piglets (kg)	Number of survivors	Weight of survivors (kg)	Voltage (V)	Size of flat electrodes (cm × cm)	Duration of electrical flow (s)
1	2	0.87; 1.38	1	0.72	75	2 × 2	15
2	0		1	0.73	65	2 × 2	15
3	0		1	0.66	75	3 × 3	15
4	0		1	0.68	90	3 × 3	15
5	0		1	0.74	65	2 × 2	30
6	0		1	0.49	75	2 × 2	30
7	3	1.48; 1.39; 1.63	1	1.48	75	2 × 2	15
8	0		1	1.58	70	2 × 2	15

#### Confirmation of death after electrocution with constant voltage

Cardiac fibrillation was recorded 1 min after the onset of the electrical flow for all five piglets, which were successfully electrocuted, while cardiac arrest was observed about 6 min later. All these piglets showed an isoelectric EEG 1–7 min after electrocution, while rhythmic breathing terminated immediately and only single gasps could be observed up to minute 3. Reflexes could not be triggered. The piglets did not show any movements and emitted no sounds. Skin irritations were mild to moderate behind the elbows.

Eight piglets survived and were raised in the clinic. They did not show any disorders besides mild to moderate burns to the skin. After current application, EEG and ECG records did not show any changes and rhythmic breathing continued. First reactions occurred after pinching the nasal septum and delayed, uncoordinated movements could be observed between minute 1 and minute 10 after the onset of the electrical flow.

#### Electrocution of anaesthetised piglets with constant amperage

Again, anaesthetised piglets (n = 39) were placed in sternal recumbency and electrodes for EEG and ECG recording were fixed. Using the high-frequency constant current electrical stunning transformer (STUN-E 514®, SCHROPP GmbH, Durach, Germany), 750 mA were defaulted. For electrocution, first flat electrodes (2 × 2 cm) were placed on both sides of the chest behind the elbow. Starting with a frequency of 50 Hz and an electrical flow lasting 15 s, the piglets survived (Table 3). The frequency was increased to 400 Hz and five piglets were successfully electrocuted. Therefore, the next four piglets were first electrically stunned (1300 mA, 400 Hz, 15 s) and afterwards electrocuted with the same setting (setting 10). However, while stunning succeeded in all piglets, cardiac fibrillation could not be achieved in the last one. To exclude the possibility that failure had been caused by body weight, the setting was tested in four piglets weighing approximately 2.0 kg (setting 11). Nonetheless, again, the last piglet survived. For the next setting (setting 12), a frequency of 730 Hz was used and tested in three piglets, but this also proved unsuccessful (Table 3).

**Table 3:** Experimental settings for electrocution of anaesthetised piglets (n = 39) using constant amperage of 750 mA

Setting	Number of successfully electrocuted piglets	Weight of electrocuted piglets (kg)	Number of survivors	Weight of survivors (kg)	Frequency (Hz)	Duration of electrical flow (s)	Type of electrodes	Position of electrodes on the chest
9	0		1	1.45	50	15	Flat	b_s^a^
10	8^b^	Between 1.35 and 1.6	1^c^	1.68	400	15	Flat	b_s
11	3	Between 2.02 and 2.10	1	2.02	400	15	Flat	b_s
12	2	2.13; 2.03	1	2.07	730	15	Flat	b_s
13	0		1	1.40	400	15/30^d^/15	Pin-shaped	b_s
14	5	Between 1.44 and 1.65	1	1.45	400	15/30^d^/15	Pin-shaped	b_s – w_s^e^
15	1	1.88	1	1.85	400	5/30^d^/5	Pin-shaped	h_h^f^ – w_s
16	0		1	1.80	400	5/30^d^/5	Pin-shaped	head^g^, h_h
17	5	Between 1.70 and 1.95	0		400	5/30^d^/5	Pin-shaped	b_s; w_s
17	6	Between 0.88 and 1.10	1^e^	0.89	400	5/30^d^/5	Pin-shaped	b_s; w_s

^a^b_s: both sides of the chest

^b^Three piglets were stunned electrically

^c^After electrical stunning

^d^Break between the electrical flows

^e^Break between the electrical flow ≤ 20 s; w_s: above the withers and the sternum

^f^h_h: above the temple and on one side of the chest (head_heart)

^g^head: above both temples

The flat electrodes were replaced by pin-shaped electrodes (Fig. 1), and were used from then on (setting 13). In one piglet, electrical current was applied twice to the heart for a duration of 15 s, with a break of 30 s in-between, which again failed. Next (setting 14), the direction for a second current application was changed, and the electrodes were placed above the sternum and the withers. Tested in six piglets, again, the setting did not lead to reproducible success (Table 3).

The following settings were taken from the standard programme of the electrical stunning transformer. First, the electrical current was applied head-to-chest for 5 s and after a break of 30 s, 5 s adjacent to the cardiac region (setting 15), using two piglets. Then the electrodes were placed above both temples for 5 s, and 30 s later to the right temple and to the left side of the chest. This setting (setting 16) was tested in one piglet. Both settings failed (Table 3).

Finally, setting 17 (Table 3) proved successful: five piglets with a body weight between 1.50 and 2.00 kg and six piglets with a body weight between 0.88 and 1.10 kg were successfully electrocuted by placing the electrodes on both sides of the chest for 5 s, and after a break of 30 s, above the sternum and the withers. Unfortunately, one further piglet survived, but in this case, the break between the two electrical flows was accidentally reduced to under 20 s.

#### Confirmation of death after electrocution with constant amperage

Cardiac fibrillation was recorded for successfully electrocuted piglets (n = 30) 1 min after the onset of the electrical flow, while cardiac arrest could be observed between minute 3 and minute 7. All these piglets showed an isoelectric EEG within 5 min after current application, while rhythmic breathing terminated already during the first minute. Single gasps could be observed up to minute 3. The piglets did not show any movements or reflex responses.

Nine piglets survived. The hearts of all but one showed short-term fibrillation but returned to sinus rhythm within 3–45 s after the onset of recording. The heart of the only piglet, where the break between the two electrical flows had been accidentally reduced, did not fibrillate. All surviving piglets breathed rhythmically from the very first minute. Four piglets did not respond to stimulations and lay motionless, while the other five piglets showed delayed, undirected movements and reactions in response to pinching the nasal septum during the observation period.

Generally, skin irritations caused by the flat electrodes were moderate to slight, while pin-shaped electrodes caused only mild alterations. Vocalisation did not occur. Surviving piglets were raised in the clinic and did not show any disorders besides the skin irritations.

#### Electrical stunning of piglets with constant current followed by electrocution

**Fig. 6:**
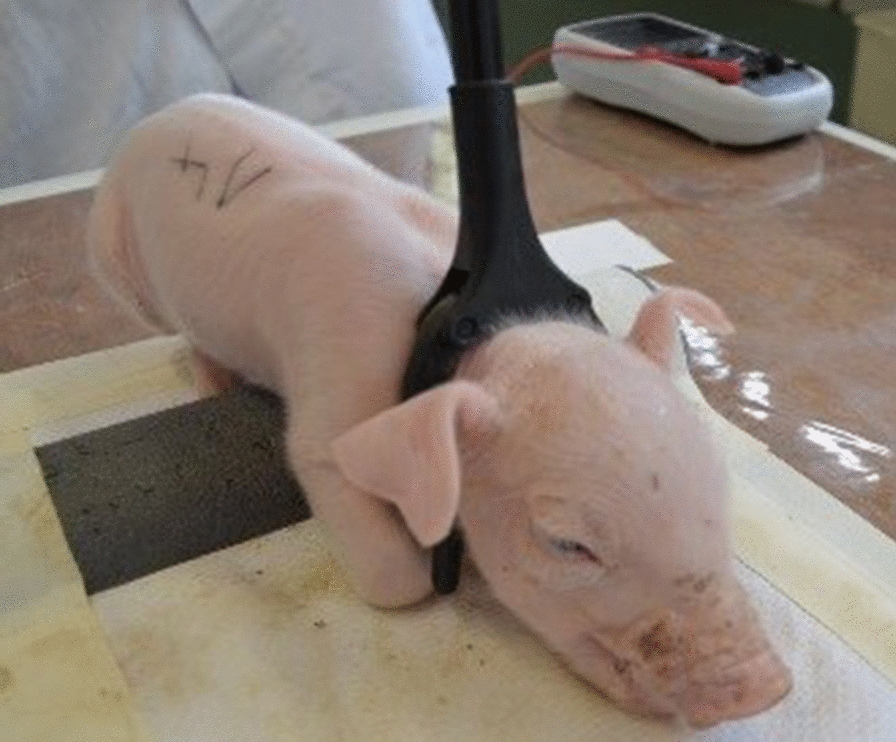
Restrained piglet using tongs for waste material before the onset of stunning

In accordance with the Council Regulation (EC) No 1099/2009 on the protection of animals, at the time of killing, a current of 1.30 A was used for stunning. Piglets were immobilised to allow a secure application of the current through the head (Fig. 6).

In accordance with the study design, stunning should be investigated after a successful setting for electrocution had been found. This meant that five piglets were successfully electrocuted one after the other with the same setting. Stunned piglets should be euthanised by using the respective setting. After setting 10 seemed to be successful, four piglets were used to examine stunning and thereafter electrocuted using setting 10. Electrocution failed in the fourth piglet (Table 3), but results of the examination of stunning are presented in this section.

First, flat electrodes were used which were placed on both sides of the head between the eyes and ears (above the temples). For stunning, frequency (400 Hz) and duration of the current flow (15 s) were maintained from the electrocution setting 10 (Table 3) to avoid time-consuming manual switching of the electrical stunning device after head stunning. All four piglets were successfully stunned.

In the subsequent step, flat electrodes were replaced by pin-shaped electrodes, the duration of the current flow being reduced to 8 s. The piglet (n = 1) was successfully stunned, but undirected paddling movements caused difficulties in testing reflexes and in positioning EEG- and ECG-electrodes.

The following setting was taken from the standard program of the electrical stunning transformer (STUN-E 514®, Freund GmbH, Paderborn, Germany). The current flow through the head lasted 10 s, during which time the frequency decreased from 500 Hz for 1 s, 200 Hz for 1 s to 50 Hz for 8 s, and the piglet was stunned effectively. However, again, paddling complicated further investigation steps. Therefore, the duration of the electrical flow was prolonged to 20 s for the examination of the reproducibility of stunning and electrocuting.

#### Confirmation of adequate unconsciousness and insensitivity after stunning

In all piglets, epileptic seizures could be observed, starting with the tonic phase, whereby the hind legs were fully stretched and the head was bent backwards while the electrodes were applied to the head. The clonic phase followed: the animals showed uncoordinated, walking movements. After prolongation of the electrical flow to 20 s, there were only a few slow movements, especially of the hind legs after the tonic seizure.

Stunning adequacy was evaluated by testing palpebral, corneal and nociceptive reflexes, which were absent in all piglets. During the time between stunning and electrocution, no rhythmic breathing could be observed. Skin irritations caused by the electrical flow were moderate above the temples. No piglet made any noises during the electrical flow or afterwards.

### Part III: electrical stunning and electrocution of 25 piglets based on the obtained data

To evaluate the reliability of this euthanasia method, 12 male and 13 female piglets with a body weight between 1 and 2 kg (average BW: 1.46 ± 0.19 kg, mean age: 3.3 ± 0.47 days) were electrically stunned and electrocuted. Using a current strength of 1.30 A and a frequency of 50 Hz for stunning, pin-shaped electrodes were placed above the temples and a current flow duration of 20 s was chosen. For electrocution, setting 17 (pin-shaped electrodes, current strength: 750 mA, frequency: 400 Hz, current flow through the heart region for 5 s from side to side, followed by a break of 30 s, and a second current flow for 5 s between withers and sternum) was used.

All piglets showed a grand mal epileptic seizure after the electrodes had been applied to the head. Thereafter, five piglets did not show any movements, while all others paddled slightly until electrocution started. Three piglets showed delayed reactions after pinching the nasal septum. No further responses to reflexes could be triggered, and no rhythmic breathing was observed.

After electrocution, death was confirmed within 3 min in all piglets by monitoring cardiac arrest and an isoelectric EEG. Rhythmic breathing was still absent. Nine piglets showed single gasps within the first minute after the onset of the electrical flow, while four piglets gasped up to minute 3. In one piglet, agonal gasping lasted for 5 min. Skin irritations on the head were moderate in 20 piglets, while five piglets showed severe burns because the layer of electrode contact gel was insufficient. On the thorax, skin irritations were absent in six piglets, while 19 piglets showed mild skin alterations. Again, no vocalisation occurred during the whole process.

## Discussion

Animal caretakers are ethically obliged to end the life of low-viability piglets to prevent them from suffering, as there is no chance of recovery through timely and humane euthanasia. When performed correctly, blunt force trauma to the head with subsequent exsanguination is an effective euthanasia method for young piglets. Nonetheless, it is less aesthetically acceptable than other alternatives [3]. Therefore, applying electrical current for stunning and cardiac arrest was investigated in order to find an alternative approach. Although euthanasia by electrocution is regarded as an efficient method that is painless for the animal [4], the effectiveness of this method in piglets with a bodyweight below 5 kg is questioned in the literature [5, 6].

To investigate electrocution independent of electrical stunning, the two-cycle method for electrical killing was chosen, a current first being applied to the head for stunning, immediately followed by the application of current across the chest to induce cardiac fibrillation and cessation of blood circulation.

The path of electrical current through the chest is a determining factor in the physiological impact of electrocution. An intuitive placing of the electrodes is not necessarily the most effective approach because the current takes the path of least electrical resistance through the tissue which is not obligatorily a straight line between the electrodes [9]. Eike et al. [10] previously demonstrated the current density in the brain simulating the electrical stunning of a slaughter pig using a finite element method computer model. Furthermore, they confirmed the best positioning of electrodes between the eyes and base of the ears on either side of the head according to Anil and McKinstry [11]. A corresponding model was constructed and current density was visualised simulating electrocution of a piglet. The computer model showed that selected sizes of the electrodes as well as the chosen position for placing the electrodes on the chest ensure that the current reaches the heart, although deviations in the current strength could be observed when comparing simulation and direct measurements in a dead piglet. One reason for deviations between the model and this experiment might be due to the fact that during the modelling, the skin was thickened unrealistically when simplifying the mesh. Hoernig et al. [12], who also used FE analysis to simulate the electrical stunning of African catfish, observed analogous discrepancies and came to similar assumptions. It must also be considered that in a dead piglet, biochemical processes have commenced, such as changes in ion mobility and cell membrane polarisation, which consequently affect conductivity [13]. Additionally, because no corresponding data are available for pigs, all types of electrical conductivity assigned to the respective tissues of the model were taken from literature which only refers to human tissue. This might contribute to the discrepancies between the model and the aforementioned experiment.

To limit the number of animals used in the animal experiments, first, the induction of cardiac fibrillation in piglets of a body weight less than 2.15 kg was investigated in anaesthetised piglets. The approved number of piglets for this part of the study was a maximum of 60 animals. In case of failure, the study should be aborted without further investigations of electrical stunning. Parameters investigated included voltage, amperage, frequency, duration of the current flow and shape of the electrodes.

In slaughter pigs, ventricular fibrillation can be induced using a minimum of 125 V and 50 Hz sine wave alternating current applied for 3 s [14]. For electrocution of swine in connection with disease control in livestock, benchmarks are 1.3 A, 250 V and 50 Hz with a minimum of 15 s current flow time; for suckling pigs a lower current strength of about 0.5 A is mentioned [15]. According to Dalziel [16], the current required to cause fibrillation is approximately proportional to body weight. The voltage of 75 V was chosen as a starting point, using the usual frequency for household electricity of 50 Hz sine wave AC and a current flow for 15 s applied by flat electrodes.

Records of the current strength 15 s after the onset of current application showed great variation between piglets. However, these indicated that piglets with a body weight of about 1.5 kg showed cardiac arrest if the gained current strength was 600–850 mA within 15 s after onset. Denicourt et al. [9] assumed that a constant current of 750 mA will be required for electrocution of lighter swine and more than 1000 mA for heavier swine. Nonetheless, they could also demonstrate that it is possible to electrocute swine effectively with a current flow of 550 mA. Accordingly, the procedure was changed and an amperage regulated circuit was used, where current strength was preset and voltage fluctuated depending on tissue resistance of the animal.

Although amperage of 750 mA was defaulted and a piglet weighing 1.45 kg was chosen, the first attempt failed and the piglet survived. Thereafter, the current frequency was raised from 50 to 400 Hz in order to disturb the cardiac T-wave to induce fibrillation by multiple high-frequency pulses. The T-wave is the part of the ECG signal that represents the ventricles beginning to relax. In the middle of the T-wave, about half of the cardiac cells are still active, while the other half return to a resting state. Electrical current delivered during this time gap leads to waves going in unpredictable paths throughout the heart and in turn leads to ventricular fibrillation. Therefore, this time period is referred to as the “vulnerable” portion of the heartbeat [17]. Nevertheless, although eight piglets (average BW 1.5 kg) were successfully electrocuted with three of them already being stunned electrically, the ninth attempt failed. A further increase in the frequency to 730 Hz did not lead to improvement.

To improve current flow by penetrating the skin layer, flat electrodes were replaced by pin-shaped electrodes. Skin resistance is the main barrier for the current flow through the body, each interruption of the current flow, e.g. caused by movement, meaning that the current must again overcome skin resistance [18]. Concerns about the extent of skin burn using the pin-shaped electrodes were unfounded although it is well known that the same current may produce thermal necrosis in the skin when the contact is made by a pinpoint electrode, but may cause no lesions when the contact area is large [19]. In the present study, skin irritations on the chest caused by the flat electrodes were mild to moderate, while pin-shaped electrodes caused only mild alterations.

In the following, the double electrical flow of the chest in two different directions (side-to-side and dorsoventral) was investigated. Both positions were already mentioned by von Mickwitz et al. [18] as causing ventricular fibrillation. Using 15 s duration of the electrical flow twice with a break of 30 s did not produce a consistent improvement. Only after reducing the duration of the electrical flow to 5 s was the procedure successful. Five piglets with a body weight ranging between 1.7 and 1.95 kg were successfully electrocuted. Since candidates for euthanasia on farms are mainly compromised piglets born with a low body weight, we also tested the setting in piglets with a body weight ranging between 0.88 and 1.08 kg. Six of these animals were also successfully electrocuted, while one piglet (0.89 kg BW) did not show ventricular fibrillation. In this particular case, the break between first and second electrical flow through the chest was accidentally reduced to below 20 s, and we assume a break of 30 s to be necessary.

The reason for the short 5 s double current application of the chest in two different directions proving successful can only be speculated. According to Kroll et al. [17], an electrical flow, which does not result in fibrillation within 5 s, will not cause fibrillation with longer duration. We assumed that the hearts of some piglets already fibrillate after the first electrical flow. These piglets´ hearts, which do not start to fibrillate or return to sinus rhythm within 30 s after the first onset of the electrical flow, need a recovery period lasting between 20 and 30 s. Thereafter, cardiac arrest could be achieved by the second electrical flow. Nonetheless, ECG was not recorded between the two electrical flows to prove this assumption.

For electrical stunning, amperage of 1.30 A was stipulated by Council Regulation (EC) No. 1099/2009 on the Protection of Animals at the time of killing. Using a constant current supply, different electrical frequencies and timespans were tested to ensure unconsciousness with only slight movements of the piglets for about 20–30 s. This time period was needed for performing reflex tests and for placing the electrodes of ECG and EEG in the piglets. In accordance with the recommendations of the Tieraerztliche Vereinigung fuer Tierschutz e.V. (TVT) (Veterinary Association for Animal Welfare) [15] in the context of euthanasia of animal groups in case of epizootic diseases, a minimum period of 8 s for the electrical flow was adopted. Regardless of the duration of the electrical flow or changes in frequency, all piglets were appropriately stunned. Spasm and contractions associated with the seizure were reduced by prolonged electrical flow. Extended application of the stunning current has already been shown to delay the onset of movements (clonic phase) because current passes down the spinal cord [20] and leads to electrical depolarisation of the spinal motoneurons to an extent that normal function does not recover. Exhaustion as a result of hypoxaemia prevents the expression of enhanced spinal reflexes and produces stillness [21].

Different indicators were used to determine unconsciousness: palpebral, corneal reflexes as well as reactions after pinching the interdigital web with surgical tweezers. All of these could not be observed after stunning and electrocution. However, delayed reactions to nose pinching were noticed irregularly after stunning and unsuccessful electrocution, which might be an indicator of recovery. However, in all stunned and successfully electrocuted piglets, rhythmic breathing was absent, which is one of the most important signs of unconsciousness after stunning [22, 23]. Additionally, righting reflex, natural blinking response, vocalisation and focused eye movement should be absent if the animal is unconscious [22], which was also confirmed.

Initially, piglets were anaesthetised with azaperone and ketamine before electrocution. Therefore, in this part of the study, reflex tests were less meaningful after the onset of the electrical flow through the chest because reactions had already been suppressed by medication. Nevertheless, an obvious indicator of effective electrocution was cessation of rhythmic breathing. Piglets with cardiac arrest showed only single gasps up to minute 3 after electrocution.

Successful electrocution was confirmed using EEG and ECG records. Measurements of the brain’s electrical function was used to objectively quantify the time of death. However, the EEG data were difficult to interpret because testing of the animal’s reflexes and the movement brought on by the gasps created interference that appeared on the EEG, which was also observed in the study by Denicourt et al. [9]. As far as a signal was recorded, an isoelectric EEG could be observed within 3 min after electrocution in all piglets in the repeatability study.

In contrast, as expected, the ECG turned out to be a reliable diagnostic tool of death in addition to the clinical observation of respiratory arrest. In all 25 piglets in the repeatability study, ventricular fibrillation was recorded 1 min after the onset of the current flow, and cardiac arrest was shown within 3 min. Agonal gasping was observed in one piglet for about 5 min, while most of the piglets lay still after 1 min.

## Conclusions

The results indicate that electrocution after electrical stunning is an appropriate method when euthanising neonatal piglets. However, the method should be tested in low-viability piglets because different variables, like dehydration and diseases, might change amperage requirements.

## Data Availability

The datasets generated during and/or analysed during the current study are available from the corresponding author on reasonable request.
